# AI use and risk disclosure by investment advisers

**DOI:** 10.3389/frai.2026.1969257

**Published:** 2026-10-14

**Authors:** Samir Chincholikar, Robin Chawla

**Affiliations:** Independent Researcher, New York City, NY, United States

**Keywords:** AI-washing, artificial intelligence, fiduciary disclosure, form ADV, generative AI, investment advisers, large language models, text classification

## Abstract

**Introduction:**

Investment advisers increasingly report using artificial intelligence (AI), but little is known about what they disclose about it in fiduciary filings. The SEC brought its first AI-washing enforcement actions in 2024, so regulators and researchers need a reproducible measure of AI disclosure. We provide large-sample evidence on AI disclosure in Form ADV Part 2A brochures and show how the estimate depends on measurement design.

**Methods:**

We drew a stratified random sample of 400 SEC-registered investment advisers from a population of 16,223 firms, crossing adviser type (private-fund vs. wealth/retail) with 2024 assets-under-management (AUM) quartile. A large language model (LLM) classified the 388 available brochures under a prespecified five-label typology: investment-process use, operational or client-service use, AI-related risk, explicit non-use, and named AI vendors. We treated the LLM as a measurement instrument and validated it three ways: same-family reproducibility, blinded cross-family recoding of 180 brochures, and human verification of 158 cases. Secondary analyses compared brochures with 283 matched firm websites and applied a screen derived from SEC enforcement orders.

**Results:**

AI use is disclosed in 23.7% of brochures (22.7% survey-weighted), while 27.6% contain AI-related risk language. The within-brochure difference is significant (McNemar *p* = 0.036). Disclosure rises from 10.4% in the smallest AUM quartile to 43.8% in the largest, and is higher among private-fund advisers. Against human coding, the composite any-use measure achieves precision of 0.76 and sensitivity of 0.81. The same corpus yields estimates of 9.9%, 13.1%, and 23.7% under alternative constructs, adviser universes, and weighting schemes. A literal keyword rule misses or mislabels roughly a quarter of cases. A raw 17.3-percentage-point brochure–website gap disappears after conditioning on document length. None of the five enforcement-screen matches was adjudicated as an unsubstantiated promotional claim.

**Discussion:**

Advisers present AI at least as often as a risk as a capability, and disclosure is concentrated among larger and private-fund advisers. AI-disclosure prevalence reflects measurement design as much as adviser behavior, so estimates should be reported with their construct, universe, weighting, and validation. The validated framework and an open reproducibility capsule provide a baseline for monitoring AI claims and AI-washing in investment management.

## Introduction

Artificial intelligence (AI) has quickly transitioned from experimental technology to operational capability claimed across financial services. Industry surveys suggest significant adoption among registered investment advisers (RIAs), with the Charles Schwab 2026 RIA and AI research study (Charles Schwab Advisor Services, 2026) finding that about 63% of surveyed firms are using or piloting AI. Form ADV Part 2A, or the brochure, is a core narrative disclosure document for investment advisers registered with the U.S. Securities and Exchange Commission (SEC) that describes the adviser’s business practices, investment strategies, conflicts, and material risks (U.S. Securities and Exchange Commission, 2019). Industry surveys provide evidence on reported AI adoption, but they do not establish what advisers disclose in fiduciary filings or how estimated disclosure prevalence depends on the way AI use is defined and measured. This distinction matters because adoption, self-reported experimentation, and formal disclosure are related but different empirical objects.

Measurement of such disclosure matters for regulatory and empirical reasons. Artificial intelligence can enter an advisory business through investment research, portfolio construction, trading, risk management, client service, operational processes or third-party technology, and these uses may raise different disclosure considerations. Regulators have begun examining whether firms represent their AI capabilities accurately, and the SEC brought its first two AI-related enforcement actions in March 2024 (U.S. Securities and Exchange Commission, 2024a,b). Before exaggerated claims can be identified, however, a reproducible way is needed to establish whether firms mention AI at all, what kind of use they disclose, whether AI is presented as a capability or a risk, and how disclosure varies across firms.

This study takes a measurement-first approach to AI disclosure among U.S. investment advisers. It asks three substantive questions. How frequently do advisers disclose AI use, and how does disclosure vary across the 2024 assets under management (AUM) and adviser-type strata? Does AI appear more often as a claimed capability or as a disclosed risk? And how does disclosure in fiduciary brochures compare with public-facing websites? These questions share a common measurement problem. An estimate of AI disclosure depends on what counts as AI use, which documents and advisers are observed, and how reliably the underlying text can be classified. We therefore treat the large language model (LLM) as a measurement instrument rather than ground truth and evaluate its classifications through same-family reproducibility, blinded cross-family recoding, and targeted human verification.

We build the study from the structured data in the SEC Form ADV Part 1, identifying 16,223 active SEC-registered advisers reporting regulatory AUM, and drawing a stratified random sample of 400 firms across adviser type and AUM quartile. We were successful in categorizing the current Form ADV Part 2A brochures for 388 firms. We employ a prespecified five-label typology that differentiates between AI use in investment process, AI use in operations or client service, AI-related risk disclosures, explicit statements of non-use, and references to named AI vendors or products. Representative positive, negative and borderline cases illustrating the framework are presented with the validation results in the section classification reproducibility and independent validation. The classification rubric and model specification were specified before outcome analysis. We evaluate reproducibility within the same family and then perform a blinded, independent cross-family recoding of 180 brochures, with validation statistics adjusted for the verification design.

Three results follow. Disclosed AI use appears in 23.7% of observed brochures, rising sharply across the 2024 AUM quartiles and occurring more frequently among private-fund advisers. AI-related risk language is at least as common as affirmative-use disclosure within the same documents. More broadly, estimated prevalence changes materially with the construct, adviser universe, weighting, and document structure used to measure it.

The difference between disclosed AI use observed in our sample and the roughly 63% adoption found in the industry survey provides useful context but does not reveal a nondisclosure rate. The two measures differ in populations, dates, reporting settings, and constructs. Survey respondents may include experimental, administrative, or internal applications as AI use even where those applications are not material to a client-facing regulatory disclosure. Brochure language may describe algorithmic practices that respondents would not themselves describe as AI. Thus, for purposes of this study, the roughly 39-percentage-point gap is taken as a benchmark divergence between reported adoption and observed fiduciary disclosure, not as evidence that a corresponding proportion of advisers have adopted AI but have not disclosed it. To measure a real within-firm uptake-disclosure gap, information matching the AI usage of the sampled advisers would be necessary.

This study makes three contributions. First, it provides a large-sample estimate of disclosed AI use among SEC-registered investment advisers and documents substantial variation across the 2024 AUM and adviser-type strata. Second, it separates AI as a claimed capability from AI as a disclosed risk and shows, using within-brochure comparisons, that risk language is at least as common as affirmative-use disclosure. Third, it shows that AI-disclosure prevalence is itself a measurement result. Estimates move materially with construct definition, sampling universe, weighting, document structure, and classification error. The classification framework is therefore evaluated rather than assumed correct, using blinded cross-family recoding and targeted human verification. The enforcement-anchored screen is retained only as a secondary methodological illustration. More generally, the paper extends the established use of financial and regulatory text as a firm-level measurement tool (Tetlock, 2007; Loughran and McDonald, 2011; Hoberg and Phillips, 2016; Cohen et al., 2020; Abis, 2022) to an emerging and policy-relevant dimension of investment-adviser disclosure.

## Institutional background and related work

### Form ADV and the fiduciary brochure

Registered investment advisers with the SEC provide information about their business on Form ADV, the Uniform Application for Investment Adviser Registration. Part 1 includes structured, machine-readable information about adviser characteristics, including legal identity, regulatory AUM, client composition, private fund activities, and disciplinary history, and is made available through the SEC’s public data infrastructure. Part 2A is the adviser’s narrative brochure and describes matters such as advisory services, investment strategies, fees, conflicts of interest, and material risks, subject to applicable filing requirements and exemptions. Part 2A is different from Part 1, which mainly provides standardized firm-level variables, in that it provides narrative descriptions of how an adviser describes its practices and risks to clients.

This distinction makes the brochure a fitting setting for studying disclosed AI use. Advisory firms may engage in AI-related activity through investment research, portfolio construction, trading, risk management, client communications, operational processes, or third-party technology. Not all internal or experimental use of AI needs to be talked about in a regulatory brochure, and so the absence of AI terminology cannot be interpreted as evidence that a firm does not use AI. In contrast, to the extent an adviser explicitly discusses AI-related capabilities, processes, vendors, or risks in its brochure, that language constitutes an observable record of how the firm discusses AI in a formal disclosure context. Thus the object of this study is disclosure, rather than the underlying technology adoption. What we measure is what is written in the advisers’ brochures, not whether a particular technology is actually deployed inside the firm.

### Enforcement of AI-washing

In March 2024, regulatory scrutiny of AI-related disclosures intensified when the SEC announced settled enforcement actions against two investment advisers for making false or misleading statements about their purported use of AI. The SEC order alleges that Delphia (USA) Inc. did not develop the capabilities it claimed to have developed to use machine learning to analyze aggregate client data in a predictive algorithmic model. Global Predictions Inc. represented itself as the “first regulated AI financial advisor” and promoted “AI-driven forecasts” that the SEC found were not substantiated as advertised. The two companies agreed to civil penalties of US$225,000 and US$175,000, respectively (U.S. Securities and Exchange Commission, 2024a,b).

These actions are relevant in a specific and limited way to the current study. They show that when firms make claims about AI capabilities that are false, misleading, or not sufficiently substantiated, this can become an enforcement problem. They also give two observable examples of the language of charged conduct. We thus use the orders to build an enforcement-based textual screen for potentially similar language in adviser disclosures and marketing materials. The screen is not designed to determine whether a firm has violated securities laws, and textual similarity alone is not evidence of misconduct. Instead, it raises a flag to check the document, and every positive case is assessed on its own merits against the underlying text. In the analysis, we call this kind of language potential AI-washing exposure language and not violations or proven cases of AI-washing.

### Related work

The paper connects three research streams, namely textual measurement, large language models as annotation tools, and the economics of AI adoption and corporate disclosure. The first treats corporate and regulatory text as data from which firm characteristics that are otherwise hard to observe can be measured. Tetlock (2007) shows that textual information can contain economically meaningful signals, and Loughran and McDonald (2011, 2016) show that financial language needs domain-specific measurement, as general-purpose linguistic categories can misclassify routine terminology in corporate disclosures. Related work uses filing text to construct measures of product-market similarity and competitive structure (Hoberg and Phillips, 2016) and finds that changes in disclosure language contain economically relevant information (Cohen et al., 2020). Similar to the current design, Abis (2022) uses language from the prospectus to differentiate between quantitative and discretionary investment management and connects the text classification with observable fund behavior. More generally, Grimmer and Stewart (2013) and Gentzkow et al. (2019) highlight that text-based measures need to be evaluated as measurement tools, not as self-validated outcomes. This study applies that approach to AI disclosure in investment-adviser brochures and centers explicit validation in the research design.

A second body of literature examines whether large language models can reliably execute classification and annotation tasks that have historically been performed by human coders. Prior work demonstrates strong performance across a variety of social-science annotation settings, such as comparisons with crowd workers, zero-shot classification, multilingual measurement, and other computational social-science applications (Gilardi et al., 2023; Törnberg, 2023; Ziems et al., 2024; Rathje et al., 2024). At the same time, there are methodological concerns about using proprietary and ever-changing models. Model outputs can vary by version, prompt, and model family, and high apparent agreement does not guarantee a classifier has captured the intended construct (Bommasani et al., 2021; Ollion et al., 2024). Such concerns are especially salient when the outputs of LLMs become variables for substantive inference rather than simply tools for exploratory analysis. Thus, from the perspective of the LLM as a measurement instrument, its performance must therefore be evaluated. The classification rubric is prespecified, the primary model and snapshot are fixed, same-family reproducibility is reported separately, and a substantial subset of documents is independently recoded by a different model family under blinded conditions. This design separates repeatability within a model family from agreement with an independent classifier.

The third strand concerns AI adoption, disclosure incentives, and potentially exaggerated technological claims. There is a growing literature on the economic implications of AI and related predictive technologies for labor demand, productivity, innovation, firm growth, and financial decisions (Agrawal et al., 2018; D’Acunto et al., 2019; Acemoglu et al., 2022; Eisfeldt et al., 2023; Noy and Zhang, 2023; Babina et al., 2024; Brynjolfsson et al., 2025). Importantly, technological adoption is difficult to observe directly in itself. For instance, Babina et al. (2024) derive firm-level AI investment from labor market information. Our setting poses a related, but different, measurement problem. The outcome of interest is not whether a firm has adopted AI, but whether it discloses AI-related activity in a fiduciary filing. The difference is important, because real adoption, self-reported adoption, and formal disclosure need not be the same.

Research on disclosure offers a framework for understanding why such differences may exist. Incentives for firms shape what information to disclose, how specific to be, and when proprietary, reputational, or regulatory concerns impact communication (Verrecchia, 2001; Healy and Palepu, 2001; Leuz and Wysocki, 2016; Blankespoor et al., 2020). More recent work considers strategic disclosure also in settings where increasingly machines are processing corporate filings (Cao et al., 2023). The possibility that claimed technological capabilities exceed substantiated capabilities is consistent with the established literature on greenwashing, where organizations can benefit from favorable claims while facing imperfect verification (Delmas and Burbano, 2011; Lyon and Montgomery, 2015). This concern has direct relevance to investment advisers in light of the SEC’s AI-related enforcement actions in 2024.

Taken together, these literatures imply that AI disclosure should be treated as a measured textual construct rather than as a direct proxy for technology adoption or misconduct. We therefore distinguish underlying AI adoption, disclosed AI use, and potentially unsubstantiated AI claims. The empirical design measures the second directly, treats the third only as a screening exercise, and evaluates the resulting disclosure measure through independent model and human coding. This structure allows the analysis to ask not only how much AI disclosure is observed, but how much that estimate changes with the choices used to construct it.

## Materials and methods

### Population frame and sampling design

The sampling frame was built from the SEC’s Form ADV Part 1 bulk filing data. We merged 27,679 unique Central Registration Depository (CRD) identifiers that appeared in filings from 2011 to 2024 and kept advisers that were active as of December 31, 2024. Of the 16,709 active SEC-registered investment advisers, 16,223 reported regulatory assets under management (AUM) and were used as the population frame for sampling.

We used a stratified random-sampling design to ensure sufficient representation across adviser business model and firm size. Advisers were divided into two broad categories, private-fund advisers and wealth/retail advisers. Within each category, firms were assigned to AUM quartiles. The two adviser categories were crossed with four AUM quartiles to produce eight sampling strata. We then selected 50 advisers from each stratum using a fixed random seed, yielding an initial sample of 400 firms. The sample was drawn and frozen before retrieval or review of any Form ADV Part 2A brochure.

We decided to allocate evenly across the eight strata to ensure sufficient observations to compare across adviser types and across the AUM distribution. Given the deliberate oversampling of strata with fewer firms, especially larger advisers, unweighted sample proportions are not treated as direct population estimates. We therefore report both design-sample estimates and survey-weighted estimates that reestablish the population distribution across the eight sampling strata.

Structured sampling variables were extracted from the year-end 2024 Part 1 population frame, and brochure retrieval, website observation, classification, and validation were performed in 2026. The temporal difference is an explicit feature and limitation of the research design. Therefore, the frozen 2024 sampling strata are defined by the adviser type and AUM variables, rather than contemporaneous measures of firm characteristics on the exact date on which the 2026 disclosures were observed. The design should therefore be interpreted as capturing current disclosure among a probability sample drawn from the year-end 2024 population of active SEC-registered advisers reporting assets under management.

The target sample size of 400 was chosen to strike a balance between the needs for document collection and validation, and precision. For proportions near 50%, a sample of 400 produces confidence intervals (CIs) of roughly ±5 percentage points (pp) at the aggregate level. Within individual strata of about 50 observations, expected confidence intervals are wider, generally on the order of ± 9 to ± 12 percentage points depending on the observed prevalence. Thus, the stratum-level estimates are interpreted mainly for broad patterns, and formal tests of the 2024 AUM-quartile gradient are performed on the combined sample.

### Form ADV Part 2A collection and analysis sample

For each sampled adviser, we attempted to obtain the current Form ADV Part 2A brochure filed with the SEC’s public filing system. Of the 400 sampled firms, 388 produced a brochure with usable text for the primary classification analysis. Eleven companies were not required to file a Part 2A brochure under the relevant filing circumstances, while one additional brochure could not be converted into usable text. All 12 excluded observations were in the wealth/retail adviser category.

The main estimand is thus AI disclosure among advisers with an observable Form ADV Part 2A brochure, rather than among every adviser in the original Part 1 sampling frame. Firms that are exempt from filing brochures are not considered to have zero AI disclosure, since the lack of a required brochure is conceptually different from a brochure that does not mention AI. As a sensitivity analysis we also examined the effect of classifying unclassified observations as nondisclosers. This does not change the substantive conclusions and changes aggregate prevalence by no more than about three percentage points.

All retrieved source documents were stored with content hashes to keep the exact text analyzed. Document processing was finalized before substantive outcome analysis. The resulting analytical corpus consists of the 388 brochures that were successfully classified.

### Classification of AI disclosure

We created and locked in a five-label classification framework to distinguish substantively different forms of AI-related disclosure prior to outcome analysis. The categories were designed to distinguish substantively different forms of AI disclosure.

The first category, *investment-process AI*, includes statements that refer to the use of AI, machine learning, or closely related computational methods in investment research, security selection, portfolio construction, trading, forecasting, or other elements of the investment process. The second category, *operations or client-service AI*, tracks the disclosed use of AI in functions such as client interaction, administrative processes, workflow automation, compliance, communications, or other noninvestment activities. The third category, *AI risk factor*, captures language identifying AI or related technologies as a source of operational, investment, cybersecurity, model, regulatory, or other risk. The fourth category, *explicit non-use*, is made up of positive statements that the adviser does not use AI or does not use AI for a specified activity. The fifth category, *named vendor*, captures mentions of a specific vendor, platform, model, or product related to AI.

These labels were used to create two composite measures. *Any AI use* equals one when a brochure includes AI in investment process, operations/client service, or a named AI vendor or product. We also report any-use prevalence after excluding the vendor category, since a vendor reference may sometimes be a weaker signal of direct firm-level use than an explicit description of an AI-enabled activity. *Any AI mention* covers any of the five labels, including risk disclosures and explicit non-use.

For each positive classification, verbatim quotes from the brochure were needed to provide support. The classification rubric was frozen before the prevalence results were calculated and included inclusion and exclusion criteria and examples for each label. The reproducibility materials include the full rubric, the prompts, the text-window specification, the model snapshot, and the classification date.

### Primary LLM classification

The 388 brochures were classified using GPT-4o as the main large language model. To minimize avoidable generation variability, a fixed model snapshot was used with the temperature set to zero. Each brochure was scored using the prespecified rubric, and the model was required to output the relevant binary labels along with supporting text for positive classifications.

The LLM was used as a measurement instrument, not as an assumed ground truth. The study therefore distinguishes primary classification from reliability and validation exercises that follow. This distinction is important because deterministic settings can enhance repeatability without establishing construct validity, and agreement between repeated outputs from the same model family does not provide the same evidence as agreement with an independently generated classification.

To assess within-family reproducibility, 60 brochures were randomly selected and independently reclassified by GPT-4o-mini using the same frozen rubric. Agreement was calculated for each label and also for the composite any-use measure. These statistics are used as measures of same-family reproducibility, not external validation.

### Independent validation

A separate validation exercise was performed using an independently operated model by another provider and model family. A total of 180 brochures were recoded with Anthropic Claude using the same frozen definitions but without access to the GPT-4o classifications. Thus, this recoding was blind to the primary labels.

The validation sample was intentionally oversampled for primary-model positives to ensure sufficient observations to estimate classification performance. We included all 123 brochures that the primary model classified as positive on the any-mention measure (any of the five labels a–e), and a fixed-seed random sample of 57 brochures chosen from the 265 corresponding primary-model negatives. So, naïve validation statistics would not reproduce the distribution of the full analytic sample, because primary positives and negatives had different probabilities of entering the verification sample.

The validation subsample is therefore a two-phase design in which the probability of entering phase two is known by construction, and metrics influenced by that design are estimated with Horvitz–Thompson inverse-probability weights (Horvitz and Thompson, 1952; Breslow and Cain, 1988). Weighted recall, agreement, and Cohen’s κ (Cohen, 1960) were calculated to represent the full classified sample. Verification weighting does not impact the precision of the any-use measure, because every brochure the primary model labeled as any-use positive falls within the censused any-mention positive stratum and therefore entered the validation set with certainty. We also report per-label agreement statistics and F1 scores.

Cross-family recoding reduces dependency on a single model architecture and provider, but it remains model-to-model validation. Agreement between two model families does not by itself establish that either is measuring the intended construct. We therefore conducted a targeted human verification exercise on 158 cases. The frame is the complete set of 60 brochures on which the two model families disagreed on any label, a stratified sample of 60 brochures on which they agreed, and 38 website cases so the rubric is assessed in both document genres. The 158-case frame was divided between two domain-knowledgeable coders, both blind to the model labels and using the same prespecified rubric. Each case received one human code. For the 120 brochure cases, disagreement cases enter the verification frame with probability one and agreement cases with a known probability below one, so model performance relative to the human coding is estimated using the corresponding two-phase verification weights. Website cases are evaluated separately. Against the human labels the composite any-use measure returns sensitivity 0.81, specificity 0.94 and precision 0.76 (95% CI 0.67–0.84). The vendor-excluded composite returns 0.76, 0.92 and 0.68. By label, precision is 0.85 for AI-related risk disclosure, 0.84 for named vendors, 0.66 for investment-process use, 0.50 for operations and client service and 0.33 for explicit non-use, while sensitivity is 1.00 for risk disclosure, 0.77 for operations, 0.75 for explicit non-use, 0.73 for named vendors and 0.71 for investment-process use. Operations and explicit non-use carry few positives, so their precision is estimated imprecisely. The 38 website cases were drawn under their own single-phase design. All 18 firms the website classifier labelled positive on any of the five labels were censused, and 20 of the 265 classified-negative sites were drawn at random under the same fixed seed, so a sampled negative carries weight 265/20 = 13.25 and a censused positive weight one. On the 38 website cases any-use precision is 0.81 and sensitivity 0.33. These website figures are estimated separately from the 120 brochure cases and are not combined with them.

### Enforcement-anchored exposure-language screen

To determine whether adviser communications contain language similar to the disclosures that the SEC has previously found in enforcement actions, we developed a narrow screening method based solely on SEC orders concerning Delphia (USA) Inc. and Global Predictions Inc. Seven textual claim patterns were distilled from the representations cited in those two actions and applied to the brochure corpus.

This procedure was intentionally designed as a high-sensitivity screen and not as a legal classifier. A document was flagged if its language was sufficiently similar to one or more of the enforcement-derived claim patterns as to require manual review. Similarity is judged on the substance of the claim by a model rater applying the frozen pattern list. No numeric similarity threshold is used, and none was preregistered. The screen’s sensitivity against the source enforcement texts was not evaluated, so its false-negative rate is unknown. The seven patterns, the screening code and the adjudication of every positive are in the reproducibility artifact. Thus, a positive screen is only potential exposure language and is not evidence of false statements, inadequate substantiation, or a securities-law violation.

The number of flagged documents was small, and each positive case was evaluated in relation to its surrounding text. The cases were then categorized according to whether the language was a false-positive textual match, an AI-related statement with supporting context or risk disclosure, or a potentially unsubstantiated promotional claim similar to the conduct described in the two enforcement orders. Results are reported in aggregate form, without identifying individual advisers.

### Website collection and cross-venue comparison

We generated a matched website sample for the sampled advisers to compare fiduciary disclosure with public-facing communication. Firm websites were collected using a standardized retrieval protocol on the homepage and on the main pages describing the firm, its investment approach, services, or technology. We collected up to eight pages per firm with the request rate limited to about one per second. Classification was made on the basis of the principal text rendered available from these pages.

Usable website observations were obtained for 283 of the 400 advisers sampled. The resulting analysis is therefore a matched cross-venue comparison, rather than a full census of the original sample. To determine whether website availability was systematically associated with observed adviser characteristics, we compared firms with usable website data to firms without such data by adviser type, AUM quartile, and AUM level.

The website measure only captures the current principal web presence of the firm at the time of collection. It is not a comprehensive coverage of dynamically rendered content that could not be retrieved, archived website versions, press releases, social media accounts, third-party profiles, or other marketing channels. Thus, the analysis is a comparison of current Form ADV Part 2A disclosure and current principal-site marketing, rather than regulatory disclosure and the full universe of the firm’s communications.

### Statistical analysis

For each classification label and composite measure, we report the observed prevalence and 95% confidence intervals. For unweighted binary proportions we use Wilson intervals (Wilson, 1927), which have better finite-sample coverage than the normal approximation, especially when the prevalence is somewhat low or when the estimates are calculated within smaller strata.

Ordered trend tests are used to test for differences by 2024 AUM quartile, with AUM quartile as an ordinal variable. We also estimate a logistic regression with disclosed any-use as the dependent variable and AUM quartile and adviser type as the main predictors. The regression is meant to assess whether the 2024 AUM-quartile gradient holds after controlling for the broad private-fund versus wealth/retail distinction. It is not interpreted causally.

Because the equal-allocation sampling design does not reflect the population distribution of the eight sampling strata, a survey-weighted estimate is also calculated. Let $N_{h}$ be the population count of stratum h in the 16,223-adviser Part 1 frame, $n_{h}$ the number of firms sampled from it and $c_{h}$ the number whose brochures were classified. Because not every adviser files a Part 2A brochure, the stratum population is deflated by the observed filing rate,


$$N_{h}^{f}=N_{h}\frac{c_{h}}{n_{h}},\;\;\;\;W_{h}=\frac{N_{h}^{f}}{\sum_{h}N_{h}^{f}},$$

where $W_{h}$ is the stratum weight. Writing ${\hat{p}}_{h}$ for the observed prevalence in stratum h , the survey-weighted estimate and its variance are


$${\hat{p}}_{w}=\sum_{h}W_{h}{\hat{p}}_{h},\;\;\;\;\hat{\mathrm{Var}}({\hat{p}}_{w})=\sum_{h}W_{h}^{2}\;\frac{{\hat{p}}_{h}(1-{\hat{p}}_{h})}{c_{h}}.$$

Sampling fractions are small, so the finite-population correction $\left(N_{h}^{f}-c_{h})/(N_{h}^{f}-1\right)$ moves the standard error (SE) only from 2.02 to 2.00 pp. It is not applied in the reported figures. The filing rate $c_{h}/n_{h}$ enters as a fixed multiplier. Resampling it together with the classified brochures in a stratum bootstrap gives 22.6% (95% CI 18.8–26.9), essentially unchanged. The regression models are unweighted. A design-weighted specification gives a coefficient on AUM quartile of 0.63 (stratum-bootstrap 95% CI 0.38–0.94) against 0.67 unweighted. Treating AUM quartile as ordinal imposes linearity, which we test by re-estimating with quartile indicators. The likelihood-ratio test does not reject the linear specification ( $\chi^{2}=0.71$ , 2 df, p=0.70 ). As a further check on sensitivity, we compute an unadjusted population-weighted estimate for the entire 16,223-firm frame. This enables us to assess the practical effects of brochure-filing exemptions on the aggregate estimate.

For the matched brochure-website sample, we compute within-firm differences in prevalence across the two venues. We calculate the uncertainty of the venue difference by a paired bootstrap that samples firms, not individual documents, so as to maintain the matched structure of the observations.

All tests are two-tailed. We apply conventional cutoffs to evaluate statistical significance, but the substantive interpretation focuses on effect size, confidence intervals, and robustness across sensitivity analyses rather than significance as such.

### Robustness and reproducibility

The empirical design includes a number of prespecified or easily replicable checks. We re-estimate any-use prevalence excluding named-vendor references, estimate prevalence using independent cross-family classifications, assess alternative treatment of brochures unavailable for classification, compare design-sample and population-weighted estimates, report same-family and cross-family agreement separately, adjudicate every enforcement-screen positive individually, and re-estimate the principal adviser-type pattern using the independent model-family recoding.

Before assessing substantive results, the sampling, classification framework, text-processing rules, prompts, model specification, and principal outcome definitions were frozen. The reproducibility package contains the frozen sample, the classification rubric, the prompts and text-window specification, the model snapshot and classification date, the enforcement-screen fingerprint, the population stratum counts, the weighting and statistical-analysis code, the validation records, the independent cross-family classifications, and the scoring code. A deterministic executable environment is provided to allow the reproduction of the reported tables, figures, confidence intervals, weighted estimates, and validation statistics from the study.

Adviser identifiers are pseudonymized, and the mapping from adviser identifiers to CRD numbers is withheld. The public package therefore supports analytical reproducibility rather than full reconstruction of the original raw-document collection. The underlying Form ADV data is publicly available from the SEC, but producing the exact source-document corpus would require access to the withheld identifier crosswalk.

## Results

### AI disclosure prevalence and form

Of the 388 observed brochures, 23.7% contain disclosure of at least one form of AI use (95% CI 19.7–28.2, Table 1). Eliminating references to specific vendors from the composite definition reduced the prevalence to 22.4% (95% CI 18.6–26.8). Only five brochures rest on a named vendor as their sole evidence of use, so the composite is not driven by mentions of third-party AI products. Because the named-vendor label is also the least reliable under cross-family validation, we report the vendor-excluded measure alongside the composite throughout. The section Robustness of the principal findings re-estimates every principal result under both definitions. The wider any-mention measure, which captured disclosed use, AI-related risk language, explicit non-use, and vendor references, was 31.7%.

**Table 1:** Typology prevalence with Wilson 95% confidence intervals (gpt-4o classifier, n=388 classified brochures).

Label	*k/n*	Prevalence (95% CI)
(a) AI in investment process	71/388	18.3% (14.8–22.5)
(b) AI in operations / client service	26/388	6.7% (4.6–9.6)
(c) AI as a disclosed risk factor	107/388	27.6% (23.4–32.2)
(d) Explicit prohibition / non-use	8/388	2.1% (1.0–4.0)
(e) Named AI vendor / product	21/388	5.4% (3.6–8.1)
Any use (a ∪ b ∪ e)	92/388	23.7% (19.7–28.2)
Any mention (a–e)	123/388	31.7% (27.3–36.5)

Labels are multi-label.

Disclosures related to risks associated with AI occurred more often than affirmative disclosures about AI use. Overall, 27.6% of the brochures had language that referenced AI, machine learning, or related technology as a source of investment, operational, cybersecurity, model, regulatory, or other risk. The pattern is that AI appears in investment-adviser brochures as a disclosed technological capability, but more often it is something to be warned about. The distinction is important because a risk-factor reference does not imply the adviser itself uses AI. Hence, risk disclosure is reported separately from the any-use measure across the analysis. Because both indicators are measured on the same brochures and may co-occur, we compare them with a paired test rather than as independent marginals. Of the 388 brochures, 77 carry both risk language and disclosed use, 30 carry risk language only, 15 disclosed use only, and 266 neither. The within-brochure difference is 3.9 percentage points (paired bootstrap 95% CI 0.5–7.2, exact McNemar p=0.036 ), and 5.2 percentage points against the vendor-excluded measure ( p=0.007 ). Risk language is therefore more common than affirmative use language within the same document, not merely in aggregate.

The survey-weighted estimate of the proportion of brochure-filing advisers that disclosed use of AI was 22.7% (95% CI 18.7–26.6, Table 2), very similar to the unweighted design-sample estimate of 23.7%. Weighting straight to the full 16,223-adviser population frame, without adjustment for brochure-filing status, produced a similar estimate of 22.3%. The small difference between these estimates implies that the prevalence rate is not greatly affected by either the equal-allocation stratified sampling design or the small number of brochure-filing exemptions.

**Table 2:** Disclosed any-use by sampling stratum.

Stratum	Filing pop. *N*	Class. *n*	Any-use (95% CI) [k/n]	Weight
Private fund—Q1	769	50	12% (6–24) [6/50]	0.049
Private fund—Q2	1,060	50	24% (14–37) [12/50]	0.068
Private fund—Q3	1,604	50	34% (22–48) [17/50]	0.103
Private fund—Q4	2,449	50	56% (42–69) [28/50]	0.157
Wealth/retail—Q1	3,271	46	8.7% (3–20) [4/46]	0.210
Wealth/retail—Q2	2,790	48	8.3% (3–20) [4/48]	0.179
Wealth/retail—Q3	2,268	48	14.6% (7–27) [7/48]	0.145
Wealth/retail—Q4	1,397	46	30.4% (19–45) [14/46]	0.089
Total (weighted)	15,606	388	22.7%	1.000

Prevalence is the share of classified brochures with Wilson 95% intervals and [k/n]. Filing population is the stratum population deflated by its brochure-filing rate, and weights are that population normalised to sum to one (see Statistical analysis).

The choice of construct matters far more than the choice of estimator. Restricting the measure to brochures containing the literal phrase “artificial intelligence” and weighting to the wealth and retail population gives 9.9%. The classifier-based any-use measure on the same population gives 13.1%, the same measure across all advisers on the design sample gives 23.7%, and the broadest any-mention measure gives 31.7%. Each step changes one decision at a time. Narrowing the construct to the literal phrase costs 3.2 percentage points among wealth and retail advisers, restricting the universe to that group costs 8.3 points, and population weighting a further 2.3. A prevalence figure for these filings is therefore only interpretable alongside the construct, universe and weighting that produced it. Against the classifier on identical text, a literal-phrase keyword rule achieves precision 0.75 and recall 0.74, so a keyword census both misses and invents roughly a quarter of cases. Reported prevalence in this literature is thus a joint statement about construct breadth, universe and weighting, not a property of the filings alone.

### Disclosure by 2024 AUM quartile and adviser type

The disclosed use of AI increased substantially across the 2024 AUM strata (Table 2, Figure 1). Among private-fund advisers, prevalence increased from 12% in the lowest AUM quartile to 24% in the second quartile, 34% in the third quartile, and 56% in the highest quartile. The corresponding estimates for wealth and retail advisers were 8.7%, 8.3%, 14.6%, and 30.4%. Hence, the pattern is not limited to one adviser category. Both groups show much higher disclosure in the higher 2024 AUM strata, although the gradient is steeper for private-fund advisers.

**Figure 1:**
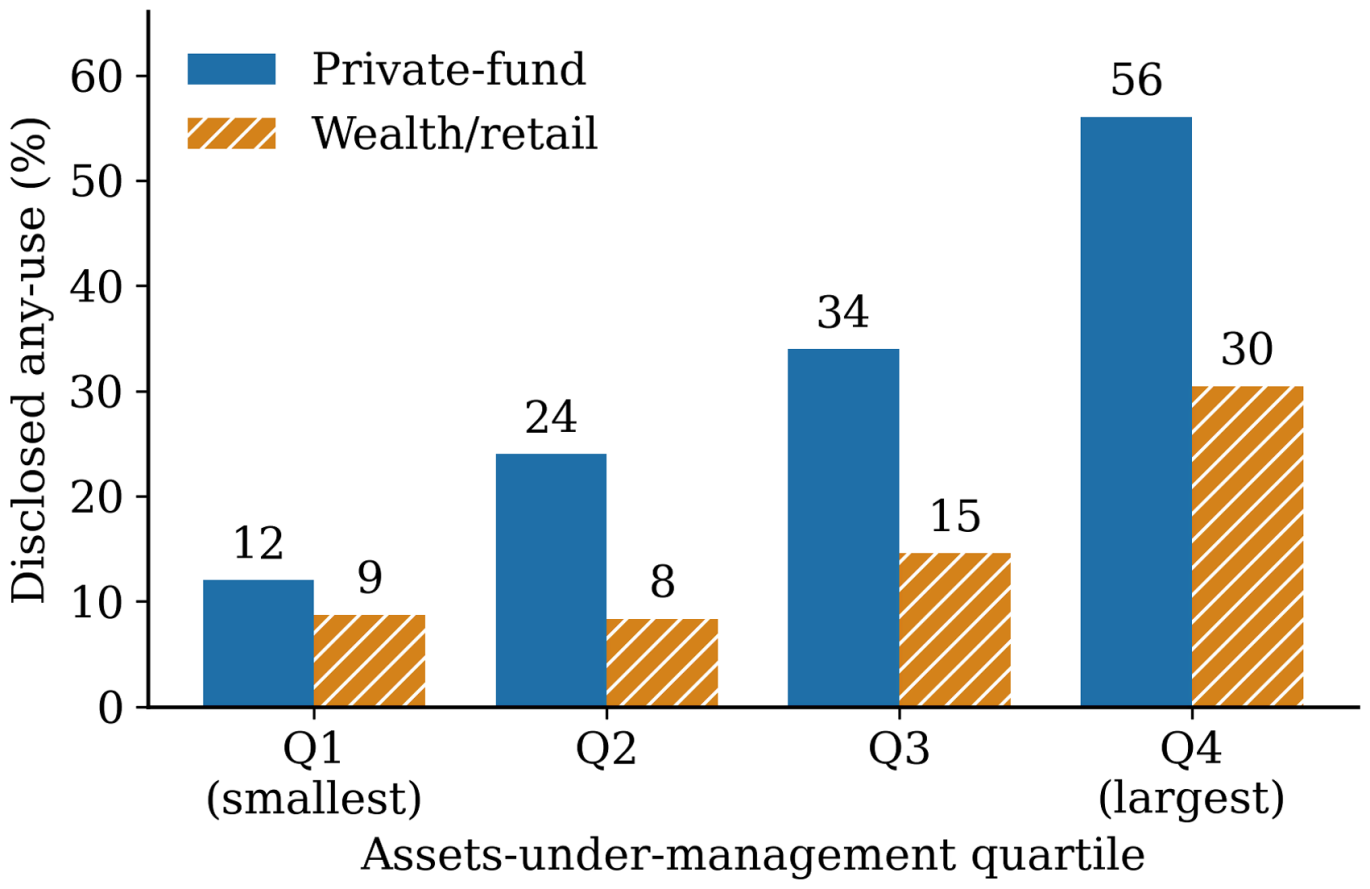
Disclosed any-use of AI by adviser type and AUM quartile. Bars show the within-stratum share of brochures disclosing any use (labels a, b or e). Private-fund advisers are solid, wealth and retail advisers hatched.

These patterns were confirmed by ordered trend tests. For private-fund advisers, the linear-by-linear trend across AUM quartiles was strongly positive ( z=4.82 , p<0.001 ). The trend was also significant for wealth and retail advisers ( z=3.00 , p=0.003 ) and the combined sample ( z=5.57 , p<0.001 ).

The same substantive conclusion was reached using a logistic regression with disclosed any-use as the dependent variable. AUM quartile was positively associated with AI disclosure after controlling for adviser type (coefficient =0.67 , SE =0.12 , p<0.001 ), and private-fund advisers were more likely to disclose AI than wealth and retail advisers conditional on 2024 AUM quartile (coefficient =1.01 , SE =0.27 , p<0.001 ). These coefficients are descriptive associations rather than causal estimates, but they show that the observed 2024 AUM-quartile gradient is not due merely to the different distribution of private-fund and wealth/retail advisers across the AUM strata.

### Classification reproducibility and independent validation

The same-family reproducibility exercise showed broad agreement, but also suggested that some substantive categories were harder to classify consistently than others. Cohen’s κ was 0.95 for AI risk disclosure and 1.00 for named-vendor references. Agreement was also high for the broader any-mention measure ( κ=0.86 ). The κ values for AI in investment processes and operations or client services were lower, at 0.61 and 0.66, respectively. Same-family agreement for the composite any-use measure was κ=0.69 .

The independent cross-family validation provided a more demanding test (Table 3). Taking the verification-sampling design into consideration, the composite any-use measure obtained a precision of 0.76, a recall of 0.84, an F1 score of 0.80, an overall agreement of 90.8%, and a Cohen’s κ of 0.74. Hence, the main classifier identified most of the independently identified positive cases and showed substantial agreement with the blinded cross-family recoding.

**Table 3:** Independent cross-family validation on the blinded 180-brochure verification sample.

Label/indicator	Prec.	Rec.	F1	% agree	κ	PABAK
(a) investment process	0.690	0.735	0.712	89.8%	0.650	0.796
(b) operations	0.808	0.677	0.737	96.1%	0.716	0.923
(c) risk factor	0.916	0.990	0.951	97.4%	0.934	0.948
(d) explicit non-use	0.875	0.467	0.609	97.7%	0.598	0.954
(e) named vendor	0.524	1.000	0.688	97.4%	0.675	0.948
Any use (a ∪ b ∪ e)	0.761	0.837	0.797	90.8%	0.738	0.816

The reference is a blind recoding by a different model family. Metrics are Horvitz–Thompson weighted to the full classified population using the two-phase verification design described in Independent validation. Precision is invariant to that weighting. PABAK is prevalence- and bias-adjusted agreement (Byrt et al., 1993).

Performance varied by classification category. AI risk disclosure showed the strongest classification performance (precision 0.92, recall 0.99, F1 0.95, agreement 97.4%, κ=0.93 ). Operations or client-service AI yielded precision of 0.81, recall of 0.68, F1 of 0.74, agreement of 96.1%, and κ=0.72 . Investment-process AI was more difficult to distinguish consistently (precision 0.69, recall 0.74, F1 0.71, agreement 89.8%, κ=0.65 ). Explicit non-use resulted in high overall agreement (97.7%) and low κ (0.60), as it was rare. Named-vendor references resulted in 97.4% agreement and κ=0.68 .

Against the 120 human-coded brochure cases the primary classifier returns any-use precision 0.76 (95% CI 0.67–0.84), sensitivity 0.81 and specificity 0.94, with per-label precision ranging from 0.33 for explicit non-use to 0.85 for risk disclosure. These estimates are weighted to the two-phase verification design. The disagreement counts reported below are descriptive tallies within the 120 reviewed cases and are not an unweighted confusion matrix for the population-level estimates.

The human coding records separate two kinds of disagreement. At the label-system level, detection disagreements occur when the model and human coder disagree over whether a retrieved passage qualifies under at least one rubric category. These arise in 21 of the 120 cases and run almost entirely in one direction. The model reads a passage as a qualifying disclosure in 20 cases where the coder does not, against 1 in the opposite direction. Boundary disagreements, where both identify qualifying AI-related disclosure but assign it to different functional categories, are more common still at 30 cases. Of these, 13 turn on investment-process use alone, 8 on operations and client service alone, and 3 on both at once, while the remaining 6 turn only on the risk, non-use or vendor labels. The per-label counts show the same pattern. Four of the five labels lean in the same direction: investment-process use has 17 model-only against 10 human-only positives, operations and client service 9 against 2, risk disclosure 13 against none, and explicit non-use 4 against 1. Only the named-vendor label leans the other way, with 3 model-only against 4 human-only. Explicit non-use carries only 8 model positives across the full sample, so its metrics rest on very few cases. The composite any-use measure is therefore more reliable than the functional split within it, and the boundary between investment-process and operational AI carries most of the residual measurement error. Table 4 gives representative human-coded cases spanning both error types. Adjusted prevalence was about 21.6% following re-estimation from the independent cross-family classifications for disclosed any-use. This estimate is within the confidence interval of the main 23.7% estimate and maintains the main adviser-type pattern. Hence, the substantive prevalence finding is not contingent on the assumption of error-free primary LLM classifications.

**Table 4:** Illustrative classification and human-verification cases.

Case	Passage (redacted)	Prim.	Indep.	Hum.	Why informative
Investment-process AI	The Advisor uses third-party artificial intelligence solutions to support investment research. These tools are used solely as…	ac	ac	ac	Explicit statement that the firm uses AI to support investment research.
Operations AI	utilizes certain artificial intelligence (“AI”) tools to enhance operational efficiency and support internal workflows, which include…	b	bd	bd	Operational efficiency and internal workflows, not investment decisions.
AI risk	Artificial Intelligence Risk. The emergence of recent technology developments in artificial intelligence and machine learning such as…	bce	bce	ce	Risk framing without evidence of adviser use.
Explicit non-use	does not use artificial intelligence (“AI”) to construct portfolios or to make recommendations. Certain third-party vendors may use AI within…	cd	cd	ac	Affirmative denial naming AI, but the coders read the passage as investment-process use with risk framing and withheld the non-use label; the boundary between a denial and a vendor’s use is where this label is contested.
Named vendor	ChatGPT and Claude (collectively, “Machine Learning Technology”) can pose risks to [firm], the Discretionary Vehicles,…	bce	bce	n/a	Vendors named inside risk language, so labels c and e co-occur.
Hard negative	Fundamental analysis is security analysis grounded in basic factors such as company earnings, balance sheet variables and management quality which are used to predict…	–	–	n/a	Conventional fundamental and technical analysis, no AI framing, no label assigned.
Detection error	Artificial Intelligence and Machine Learning. Recent technological advances in artificial intelligence and machine learning technologies (collectively, “AI Technologies”), as well as the rapid growth and widespread use thereof, have the potential to disrupt the markets in which we operate…	ac	c	ac	Generic AI-risk passage; models differ on whether firm use is established.
Boundary error	AI in connection with its investment, research, operational or decision-support processes. For purposes of this disclosure, AI…	ac	abcd	ac	A single sentence spans investment, research and operational use.

Passages are quoted verbatim with the filing firm’s name, its acronym and personal names redacted. Prim. and Indep. are the labels assigned by the two model families, and Hum. is the human-coded label where available. A dash denotes no label, and n/a denotes a case outside the human-verification frame. A detection disagreement is disagreement over whether a passage qualifies at all; a boundary disagreement is over which functional category applies.

Here (a) is investment-process AI, (b) operations or client-service AI, (c) AI as a disclosed risk factor, (d) explicit prohibition or non-use, and (e) a named AI vendor or product. A case can carry several labels at once, so ac means labels (a) and (c) together.

### Enforcement-anchored exposure-language screen

Of the 388 brochures in the classified sample, five (1.3% of the classified sample, 95% CI 0.6–3.0) were identified for case-level review using the enforcement-derived screen. The screen was designed to identify textual resemblance, not misconduct, so all five positive cases were examined individually in context.

Two were false-positive textual matches in which the relevant language did not constitute an affirmative promotional claim regarding the adviser’s AI capabilities. The other three had actual references to AI or machine-learning activities, but also contained contextual or risk-related language that bolstered or qualified the statements. None was adjudicated as an unsubstantiated promotional claim of the nature alleged in the SEC’s two enforcement actions.

The resulting estimate should not be taken as the prevalence of AI-washing. The screen captures only language that appears to be a narrow set of claim patterns that come from two enforcement cases, and the number of positives is small. Rather, the result suggests that language similar to the charged representations was rare in the sampled fiduciary brochures and that the screening procedure did not produce a single instance that could be classified as an unsubstantiated promotional claim based on the text available.

### Fiduciary disclosure and public-facing marketing

Current website observations were obtained and usable for 283 sampled advisers. Within this matched sample, disclosed AI was found in 23.0% of Form ADV Part 2A brochures, but only in 5.7% of current principal-site marketing materials (Table 5, Panel B, and Figure 2). The paired bootstrap 95% confidence interval for the 17.3-point difference is 12.0–22.6.

**Table 5:** Robustness of the principal results.

Panel A. Vendor-excluded definition		
Result	Incl. vendor (a ∪ b ∪ e)	Excl. vendor (a ∪ b)
Overall prevalence	23.7% (19.7–28.2)	22.4% (18.6–26.8)
AUM quartile 1	10.4%	8.3%
AUM quartile 2	16.3%	15.3%
AUM quartile 3	24.5%	23.5%
AUM quartile 4	43.8%	42.7%
Private-fund advisers	31.5%	29.0%
Wealth and retail advisers	15.4%	15.4%
Survey-weighted (brochure filers)	22.7%	21.8%
Weighted to full 16,223-adviser frame	22.3%	21.5%
Logistic coefficient, AUM quartile	+0.67 ( p<0.001 )	+0.72 ( p<0.001 )
Logistic coefficient, private fund	+1.01 ( p<0.001 )	+0.89 ( p<0.001 )
Risk language minus disclosed use	+3.9 pp. ( p=0.036 )	+5.2 pp. ( p=0.007 )

Panel B. Cross-venue comparison, matched firms (*n*=283)		
Measure	Brochure vs. website	Website - brochure
Any-use disclosure (a ∪ b ∪ e)	23.0% vs. 5.7%	−17.3 pp. ( −22.6 , −12.0 )
Any-use, vendor-excluded (a ∪ b)	21.9% vs. 5.7%	−16.3 pp. ( −21.6 , −11.3 )
Exposure-language screen (F1–F7)	0.7% vs. 0.7%	0.0 pp. ( −1.4 , +1.4 )

Panel C. Website coverage		
Group	Usable/total	Coverage
Private-fund advisers	140/200	70.0%
Wealth and retail advisers	143/200	71.5%
AUM quartiles 1 to 4	73, 65, 71, 74/100	73.0, 65.0, 71.0, 74.0%
Median regulatory AUM	covered US $419 M vs. uncovered US $357 M	

Panel A re-estimates each result with the composite defined as investment-process or operations AI only (a ∪ b), dropping the named-vendor label. Panel B reports the matched cross-venue comparison, with paired bootstrap 95% intervals. Panel C reports website coverage, where no coverage difference is significant (type p=0.83 , quartile p=0.50 , AUM p=0.46 ).

**Figure 2:**
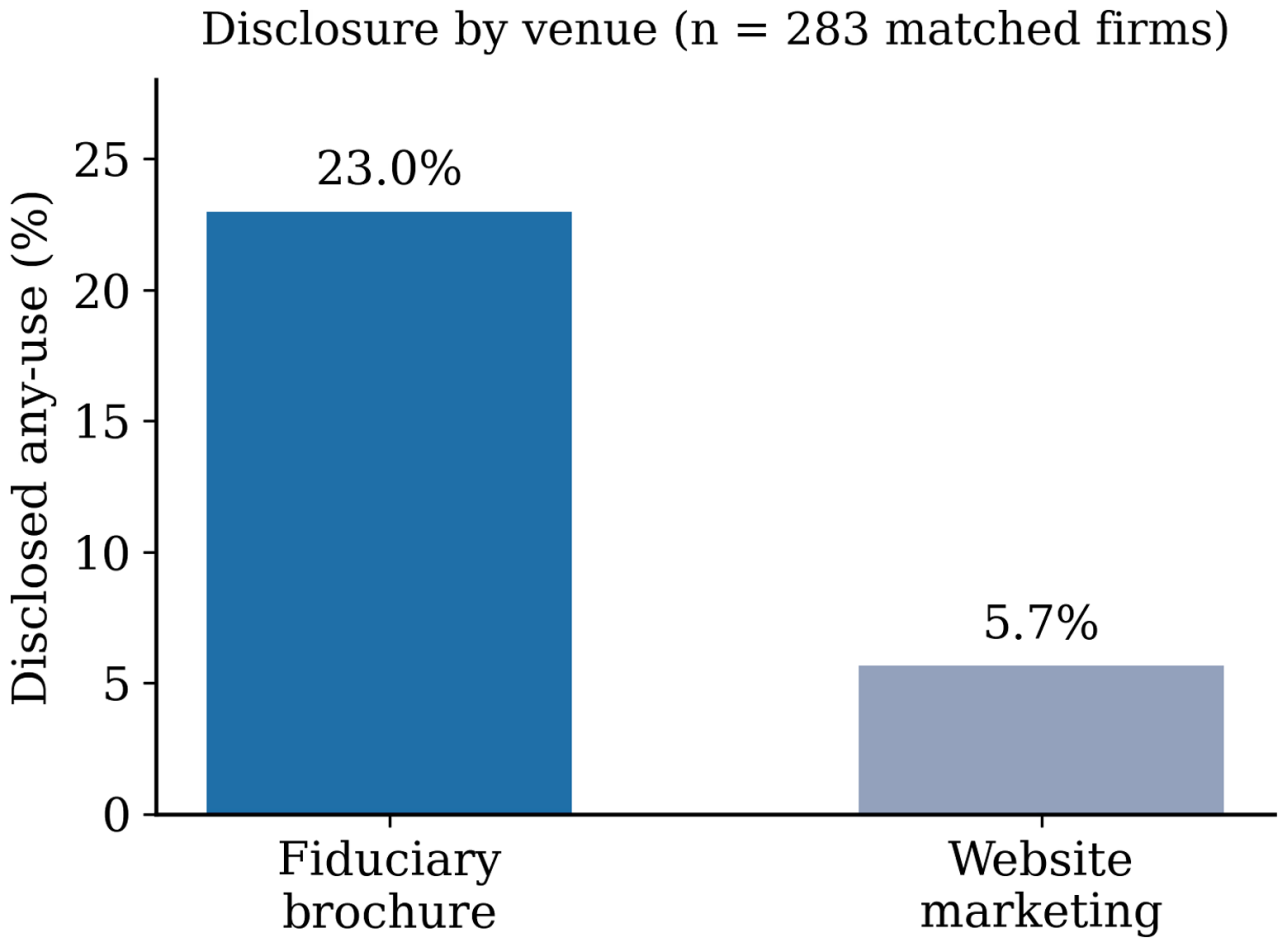
Disclosed any-use of AI by venue for the 283 firms observed in both venues. The matched brochures carry 7.3 times the text of the matched websites, and the venue difference does not survive conditioning on document length (see Fiduciary disclosure and public-facing marketing).

This pattern is supported by the underlying matched observations. Fifty-eight firms disclosed AI usage in their brochure but not on the observed website, with only nine disclosing the opposite pattern. Thus, among firms with both venues visible, disclosure in the brochure only was much more common than disclosure on the website only.

This raw contrast, however, is confounded by how much text each venue provides. The matched brochures carry 7.3 times the characters of the matched website corpora (26.2 million against 3.6 million, with medians of 62,412 against 6,769). In a stacked model of document-level disclosure with standard errors clustered by firm, the brochure indicator is strongly positive on its own (coefficient 1.61, p<0.001 ) but is no longer distinguishable from zero once log document length is included (coefficient −0.54 , p=0.17 ), while length itself is strongly positive ( 1.14 , p<0.001 ). Per 100,000 characters, websites in fact return slightly more detections than brochures (0.45 against 0.25), and within the overlapping length band the two venues are close (11.4% against 8.9%). Because brochure and website lengths barely overlap, venue and length cannot be separated in this design. We therefore report the venue contrast as a description of what a reader encounters in each document type, and not as evidence that advisers choose to discuss AI more candidly in fiduciary filings than in marketing. The same caution applies to any binary disclosure indicator compared across documents of unequal length.

The enforcement-anchored exposure-language measure was rare in both venues, at about 0.7% of observations in each. The paired bootstrap estimate of the difference was essentially zero (95% CI approximately −1.4 to 1.4 pp). There is no evidence in the matched sample, therefore, that the narrow enforcement-derived language patterns were more prevalent on current websites than in fiduciary brochures.

There was also little evidence of systematic selection on observed sampling characteristics in website availability (Table 5, Panel C). Coverage did not differ significantly by adviser type ( p=0.83 ) or AUM quartile ( p=0.50 ). The median AUM for firms with usable website observations was approximately US$419 million and US$357 million for those without, a difference that was not statistically significant ( p=0.46 ). Availability is also unrelated to the outcome being compared. Brochure-disclosed any-use is 23.0% among the 283 firms with usable website text and 25.7% among the 105 without ( p=0.67 ), with the same null for the vendor-excluded measure ( p=0.79 ) and for risk language ( p=0.25 ). None of these comparisons establish that missingness of websites is random conditional on all unobserved characteristics, but they do alleviate concerns that the matched sample is mechanically concentrated among one of the principal adviser categories considered in the study, or selected on the disclosure outcome itself.

Targeted human verification indicates that classification is less sensitive on website text than on brochures. Website any-use precision is 0.81 and sensitivity 0.33. The observed website prevalence should therefore be interpreted cautiously, because the classifier may miss a substantial share of qualifying website passages. This measurement difference further limits interpretation of the raw prevalence gap between brochures and websites.

### Robustness of the principal findings

Table 5, Panel A, re-estimates every principal result under the vendor-excluded definition. Excluding vendor-only references reduces prevalence modestly, from 23.7 to 22.4%, and leaves the 2024 AUM-quartile gradient, the adviser-type association, both weighting schemes, the paired risk comparison and the cross-venue pattern unchanged. The gradient is marginally steeper under the stricter definition. Re-estimation from the independent cross-family classifications yields about 21.6%, within the primary confidence interval, and the alternative treatment of the 12 unclassified brochures changes the aggregate only modestly. The three weighting schemes lie within 1.4 percentage points of one another.

The 2024 frame against 2026 observations is testable rather than merely acknowledged. Across a two-year interval 77.0% of sampled advisers remain in the same AUM quartile (Spearman ρ=0.89 ), and re-estimating the logistic model on deliberately lagged strata leaves the gradient intact. The coefficient on AUM quartile is 0.67 on the 2024 stratum, 0.52 on strata rebuilt from 2022 assets and 0.79 from 2020 assets, each at p<0.001 .

## Discussion

### AI as a disclosed capability and a disclosed risk

AI-related risk language appears in 27.6% of brochures, a higher rate than the any-use measure, and the difference holds within the same documents. Artificial intelligence, machine learning and generative AI can be described in a brochure precisely because those technologies generate cybersecurity, investment, operational, model, legal or regulatory risks. Any reference to AI therefore overstates disclosed use, which matters most for automated text analysis, where keyword measures combine conceptually different statements into a single indicator. The five-label framework addresses this by separating investment-process use, operations or client-service use, risk disclosure, explicit non-use and named-vendor references. The prominence of caution is consistent with the institutional function of Form ADV Part 2A, which exists to describe material practices, conflicts and risks rather than to promote, though the cross-sectional design cannot establish why individual advisers chose particular language.

### Measuring disclosed AI use

The central measurement result is that there is no construct-free prevalence of AI disclosure. The same corpus produces materially different estimates as the definition, adviser universe, and weighting scheme change. Narrowing the measure to the literal phrase “artificial intelligence,” restricting the universe to wealth and retail advisers, and then broadening the construct move prevalence between 9.9%, 13.1%, and 23.7%. A literal-phrase rule also has only 0.75 precision and 0.74 recall against the classifier, showing that simple keyword counts both omit qualifying cases and include nonequivalent ones. The same issue appears within the taxonomy. Named-vendor references provide weaker evidence of adviser use than descriptions of an AI-enabled process, while human verification shows that most residual disagreement concerns the boundary between investment-process and operational AI rather than the composite any-use measure itself (Table 5 reports every principal result under the vendor-excluded definition). Document structure adds another layer. Brochures contain far more searchable text than websites, and the apparent venue difference disappears after conditioning on document length. The appropriate object of interpretation is therefore the validated composite disclosure measure, with narrower functional categories and cross-venue comparisons treated more cautiously.

The outcome is disclosed use, not underlying adoption. Formal disclosure sits far below survey-reported adoption, roughly 23% of brochures against the 63% of advisers reported to be using or piloting AI, but the two instruments measure different constructs on different populations at different dates. An adviser may use AI administratively without disclosing it, and a brochure may describe algorithmic or machine-learning methods that a survey respondent would not call AI. The gap between survey reports and disclosure is a benchmark divergence, not a nondisclosure rate.

### Adviser heterogeneity

Disclosed AI use rises steeply across the 2024 AUM strata, from 12 to 56% across AUM quartiles among private-fund advisers and from roughly 9 to 30.4% among wealth and retail advisers, and the gradient survives the addition of adviser type to the regression. Private-fund advisers disclose more often conditional on 2024 AUM quartile, which is consistent with systematic investment approaches, alternative data and specialized technology infrastructure generating more disclosure-relevant references. Several mechanisms could produce these patterns, including technical and organizational resources, the breadth of activities in which AI can be embedded, and compliance capacity for documenting emerging technologies in filings. The design cannot separate them, and the associations are not causal. What the result establishes is that AI disclosure is unevenly distributed across the adviser universe rather than diffuse.

### Cross-venue and enforcement evidence

Among the 283 advisers observed in both venues, 23.0% of brochures and 5.7% of websites contain disclosed AI use, and 58 firms disclose in the brochure only against nine in the website only. It is tempting to read this as evidence that AI language is not primarily promotional, but the design cannot support that reading, because the venue indicator is indistinguishable from zero once document length is included and the two length distributions barely overlap. We restrict the claim to what is observed. Raw prevalence differs across the two corpora, with brochures higher.

The enforcement-anchored screen triggered on five of the 388 brochures. None was adjudicated as an unsubstantiated promotional claim of the type charged in the two orders. The contribution is methodological rather than substantive. Enforcement orders can be distilled into observable claim patterns that flag documents for review, but a textual match is not misconduct, and the screen rests on two actions. We therefore report no estimate of AI-washing prevalence.

## Limitations and future research

### Limitations

Four limitations qualify the findings. First, the study measures disclosed AI use rather than underlying adoption. The estimates are properties of documents, not of the technologies firms deploy.

Second, the disclosure observations are not contemporaneous with the sampling frame. Adviser type and AUM quartile were determined from the 2024 year-end Form ADV Part 1 population, while brochure retrieval, website collection and classification occurred in 2026. We therefore describe the size results as associations with sampling-stratum characteristics rather than with contemporaneous adviser size. The section Robustness of the principal findings reports the empirical magnitude of the problem and a lagged-stratum falsification test.

Third, measurement error remains after validation. Against the 120 human-coded brochure cases, any-use precision is 0.76 (95% CI 0.67–0.84), sensitivity is 0.81, and specificity is 0.94. The composite measure therefore retains meaningful classification error even after validation, with model-positive/coder-negative passages remaining the larger source of uncertainty. Agreement is consistently weaker for the investment-process and operations/client-service categories than for AI-related risk disclosure, so the boundary between functional categories carries more uncertainty than the composite any-use measure. The human verification also shares the classifier’s retrieval step. Coders judge the same keyword-selected passages the model was shown, not the full brochure, so the exercise bounds error in assigning labels to retrieved text and not error in retrieving it. Weighting cannot remove this error, nor correct for changes in firm characteristics after the sampling date.

Fourth, the design is cross-sectional and specific to U. S. SEC-registered advisers, and both secondary analyses are bounded. The website comparison covers 283 firms with usable current principal-site content and excludes dynamically rendered pages, archived versions, advertisements, social media and press releases, so cross-venue conclusions should not be generalized to all adviser marketing. The enforcement-anchored analysis rests on two enforcement actions and five positives, so it identifies text for review rather than estimating the prevalence of enforcement-relevant language. The findings should not be generalized to state-registered advisers, broker-dealers, banks, or firms in other countries, and the study cannot say whether the observed differences are caused by size, business model, regulatory scrutiny or technological capacity.

### Future research

Three extensions follow directly. A longitudinal panel of adviser brochures would separate persistent firm characteristics from changes in disclosure behavior, and show whether the diffusion of generative AI reaches formal filings with a lag. Matched firm-level data on technology adoption would allow an adoption-disclosure relationship to be estimated rather than benchmarked against an unmatched survey. And widening the set of venues beyond brochures and principal websites, to advertisements, social media, press releases and investor presentations, would test whether AI claims emerge in promotional communication before entering formal disclosure.

## Conclusion

Disclosed AI use appears in roughly a quarter of U. S. investment-adviser fiduciary brochures, and it is unevenly distributed. Prevalence rises steeply across the 2024 AUM strata and is higher among private-fund than wealth and retail advisers. AI-related risk language is at least as common as affirmative use language within the same documents, so AI enters these filings as a risk to be disclosed as much as a capability to be described. The headline figure depends materially on how the construct is defined, which advisers are counted, and how much text each document contains. The same corpus supports estimates from roughly 10 to 24%, and a binary indicator rises with document length. Prevalence in this literature is therefore a property of a measurement design as much as of the filings it is drawn from.

## Data Availability

All data and code required to reproduce the study are openly available in a deterministic Code Ocean capsule (Chincholikar and Chawla, 2026) (https://doi.org/10.24433/CO.3404788.v2, MIT license). Within the capsule, the/data directory contains the frozen stratified sample (data/sample.csv) covering 400 sampled firms and 388 classified, the classifier label sets for the primary gpt-4o labels (data/labels_primary.csv), the blinded cross-family recoding (data/labels_independent.csv) and the same-family subsample (data/labels_secondary_sub.csv), the human verification records for the brochure and website cases with the two-phase frame definition that weights them (data/human_verification/), the population stratum counts (data/population_strata.csv), the venue and exposure tables (data/venue/), and the classification rubric and enforcement fingerprint (data/prompts/). The analysis, weighting, and validation code is in/code and pre-computed outputs in /results. The capsule regenerates every reported number, table, and figure from the frozen data, including the illustrative cases in Table 4, whose label codes are derived at run time from the frozen label files, and both published figures at their typeset resolution. The source repository is openly available at https://github.com/samirrc2/AI-Use-and-Risk-Disclosure. Adviser identifiers are pseudonymized and only aggregate results are reported. The crosswalk to CRD identifiers is withheld. The underlying SEC Form ADV Part 1 data, Part 2A brochures, and enforcement releases IA-6573 and IA-6574 are publicly available through the SEC’s IAPD system.
